# Proposed classification of adenomyosis in Infertile women to simplify management options undergoing ART

**DOI:** 10.5935/1518-0557.20240015

**Published:** 2024

**Authors:** Sunita Tandulwadkar, Sneha Mishra, Swapnil Langde, Mily Pandey, Rashmika Gandhi

**Affiliations:** 1 Ruby Hall Clinic, Pune, India

**Keywords:** adenomyosis, infertility, adenomyosis classification, pregnancy, IVF

## Abstract

**Objective:**

Adenomyosis associated with subfertility is a situation of a dilemma for the treating clinician as the treatment is highly controversial and there remains an overall lack of consensus regarding the value of conservative surgery with or without medical management to improve reproductive out-comes. Hence we proposed this classification based on mapping of the size of adenomyoma, its location, distance from the endometrial cavity, and any associated endometriosis by studying 100 women with adenomyosis undergoing IVF.

**Methods:**

We did a prospective study over 2 years in 100 women with adenomyosis who underwent IVF. They were classified into 4 categories based on our management-based proposed classification and the pregnancy outcomes were studied in each group.

**Results:**

According to our classification, 56% of women belonged to grade 1, 24% to grade 2, 8% to grade 3, and 12% to Grade 4 Adenomyosis. The Pregnancy rates were 71% in Grade 1, 66% with Medical management, and 33% with surgical management in Grade 2, Grade 3 were offered surrogacy, and 66% in Grade 4 Adenomyosis.

**Conclusions:**

Our classification is simple and allows cost-effective management based on the location and ex-tent of the disease with the help of ultrasonography.

## INTRODUCTION

Adenomyosis associated with infertility is still an enigma for the treating clinician. Women with adenomyosis have lower implantation rate per embryo transfer, lower clinical pregnancy rate and higher spontaneous abortion rate as compared to women without adenomyosis.

Although the exact mechanism behind the relationship between adenomyosis and infertility is still unclear, a number of factors has been proposed and focus on four putative pathways: (i) Intrauterine abnormalities and increased uterine peristalsis causing abnormal utero-tubal sperm and embryo transport. Intrauterine anatomical distortion caused by uterine hyper peristalsis and inflammation-induced adnexal adhesion may block the tubal ostia and potentially impair sperm migration and embryo transport (Harada *et al*., 2016; Brosens *et al*., 2004). (ii) Abnormal endometrial steroid metabolism, increased inflammatory response, and increased intrauterine oxidative stress environment leading to altered endometrial function and receptivity (Brosens *et al*., 2004). The increased density of macrophages increases inflammatory response of the endometrium and release of reactive oxygen species that are thought to be embryotoxic. (iii) Impairment of implantation may result from inflammation, a lack of adequate expression of adhesion molecules (integrins), reduced expression of implantation markers, such as leukemia inhibitory factor (LIF), and altered function of the gene for embryonic development (HOXA10) (Emge, 1962) (iv) Occurrence of chronic endometritis (CE) resulting from intrauterine microbial infection may be associated with negative fertility outcome in women with adenomyosis.

The prevalence of adenomyosis in a population of infertile women ranges between 7% and 27% (Dueholm & Aagaard, 2018). If women with adenomyosis undergo an in vitro fertilization treatment, there will be a significant reduction in clinical pregnancy and delivery rates as compared to women without adenomyosis (a clinical pregnancy rate of 41% *vs*. 50% and of 26.8% *vs*. 37.1%) (Mavrelos *et al*., 2017) Miscarriage occurs in 32% women with adenomyosis and 14% in those without adenomyosis (Vercellini *et al*., 2014). Even with maximum possible cytoreduction spontaneous pregnancy rate in women with adenomyosis is 60%. In cases where only partial excision is done, these results decreased to 47%. It should be known that none of these data are from randomized trials, but mostly from small and retrospective series (Mavrelos *et al*., 2017).

Adenomyosis associated with subfertility is a situation of a dilemma for the treating clinician as the treatment is highly controversial and there remains an overall lack of consensus regarding the value of conservative surgery with or without medical management to improve reproductive outcomes Leyendecker *et al*. (2015). Also, there is no standardization on when and whom to operate and surgical techniques to be used. Existing literature demonstrates increased miscarriage rates and poor pregnancy outcomes in such women depending on the extent of abnormal uterine myometrium with different cell density and immunohistochemistry as compared to normal uterine myometrium.

There have been many attempts to classify adenomyosis based on Histopathology, Ultrasound and MRI features and MUSA (Morphological Uterine sonographic assessment) being the most accepted one. But none of the classifications have been able to prognosticate the treatment outcomes in women with adenomyosis with infertility. This necessitates the need for a more simple and stratified approach to adenomyosis classification for universal and standardized management.

Hence, we proposed this classification based on mapping of the size of adenomyoma, its location, distance from endometrial cavity and any associated endometriosis by studying 100 women with adenomyosis undergoing IVF.

## Our proposed classification

**Figure 1:**
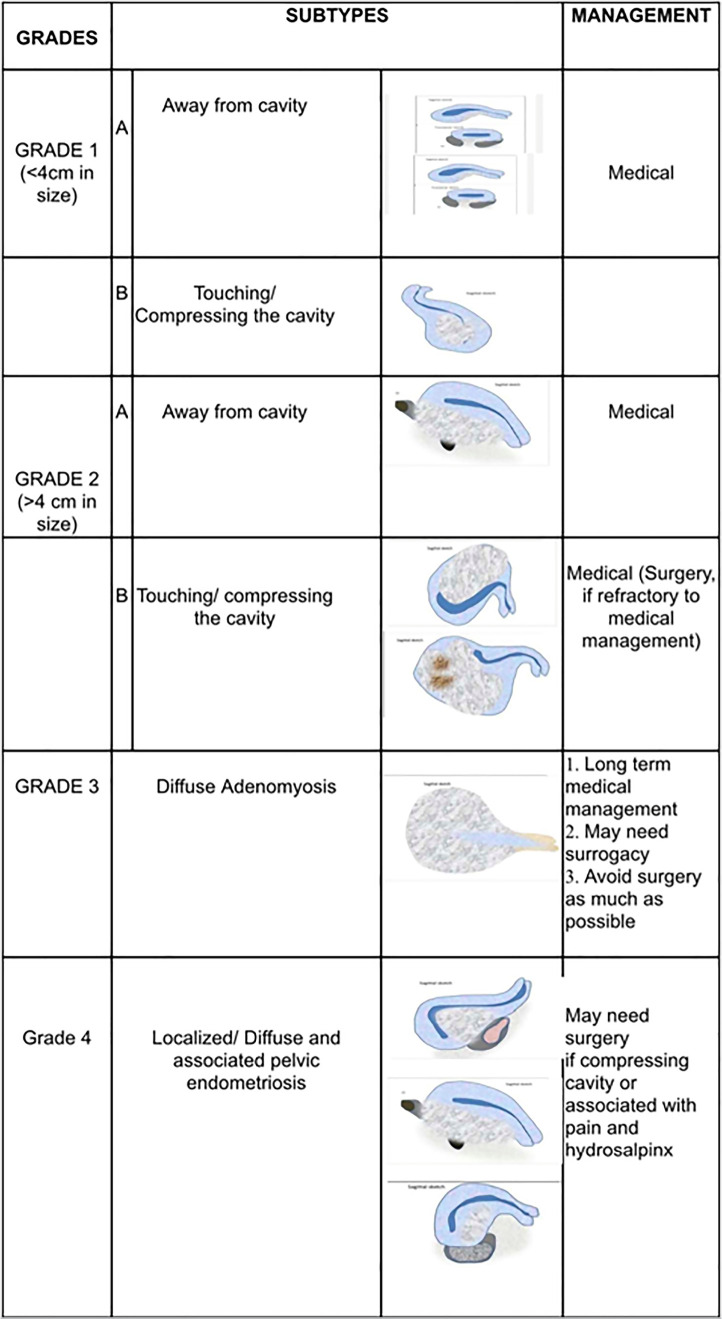
Proposed adenomyosis classification.

Infertile women presenting to IVF OPD underwent transvaginal ultrasound as a part of routine infertility workup. The sonography was done by a single experienced radiologist using a transvaginal probe of 8 MHz and women with ultrasound features of adenomyosis as per MUSA adenomyosis criteria were taken up in the study group. MUSA adenomyosis ultrasound features include: Asymmetrical thickening, fan shape shadowing, cyst, hyperechoic island, echogenic sub endometrial lines and buds, trans lesion vascularity, irregular junctional zone, and interrupted junctional zone. Adenomyosis was further classified based on the size and location of adenomyoma and its relation to endometrial cavity, into 4 categories (Figure 1):

**Grade 1:** Adenomyoma less then 4cms in size. It is further subclassified into A and B

Grade 1A - away from the cavity

Grade 1B - touching or compressing the endometrial cavity.

**Grade 2:** Adenomyoma greater than 4cms in size

Grade 2A - Away from the cavity

Grade 2B - Touching/compressing the cavity.

**Grade 3:** Diffuse adenomyosis.

**Grade 4:** Localized/diffuse disease with associated pelvic endometriosis.

4 cm criteria: As most criteria’s for fibroid removal have 4 cm as cut off, hence we are considering the same size for adenomyosis in our study.

The best way to study the impact of adenomyosis on implantation is to study the adenomyosis patients requiring IVF. The question that “Does the presence of adenomyosis have deleterious effect on fertility and if so, does Pre-embryo transfer therapy will improve the results?” is still an enigma.

## MATERIAL AND METHODS

Based on the proposed classification, a prospective cohort study was conducted in department of IVF and Endoscopy over a period of 2 years from July 20 - July 22. Study population included women with uterine adenomyosis undergoing IVF.

### The inclusion criterion was:

Women with adenomyosis undergoing IVF.

### The following women were excluded from the study:

Women with evidence of uterine fibroid or any other structural anomaly of uterus.

Women with male factor infertility

3. Women with Ovulatory dysfunction.

A sample size of convenience of 100 women was taken. A survey sheet was prepared to obtain all the necessary information. All the information obtained was recorded in the predetermined format.

We took a sample size of convenience of 100 women with adenomyosis who were undergoing IVF with long agonist protocol and frozen embryo transfer.

1. Women with Grade 1A and 1B adenomyosis were treated with medical management in the form of 6 months of GnRH injection followed by a frozen embryo transfer in HRT cycle.

2. Patients with Grade 2A were offered medical management with GnRH agonist injection (1-2 doses) and Grade 2B or those who were refractory to medical management with GnRH were taken up for surgery (adenomyomectomy). Refractory to medical management was defined as decrease in size of adenomyosis by less than 20% of original volume.

3.Women with diffuse adenomyosis in Grade 3 who failed medical management were offered surrogacy.

4.Women in Grade 4 with adenomyosis with endometriosis with hydrosalpinx were offered surgery in the form of endometrioma excision with adhesiolysis and hydrosalpinx excision. For adenomyosis, surgery was offered for local lesions followed by GnRH and in diffuse cases, 1-2 doses of GnRH 11.25 mg were given.

### Statistical analysis

A simple grid was prepared to collate the data in the survey sheet and proportion of responses for each question was calculated.

### Ethical approval

The study was approved by institutional ethics committee.

## RESULTS

A total of 100 women with adenomyosis who underwent IVF were studied. The mean age of participants was 32 years. Most of the subjects in the study group belonged to upper and upper middle socioeconomic status by modified Kuppuswami scale. 56% women belonged to grade 1, 24% to grade 2, 8% to grade 3 and 12 % to Grade 4 Adenomyosis according to our classification (Table 1).

**Table 1:** Management and pregnancy outcomes.

GRADE	Sample size	Medical	Medical+Surgical (Adenomyomectomy)	Medical+Surgical (Adhesiolysis+hydrosalpinx excision)	Pregnancy rate
**1**	56	56	0	0	40 (71.4%)
**2**	24	16	8	0	Medical - 66.6% Surgery - 33.3%
**3**	8	8 (Failed)	0	0	Offered Surrogacy
**4**	12	5	3	4	66.6%

Decrease in volume of adenomyoma sufficient enough to keep the compression away from cavity was the criteria to decide results of medical management before proceeding with FET.

1. All the women with Grade 1 adenomyosis (n=56) were given medical management in the form of injection GnRH 11.25 mg for 3 months and then followed by Frozen embryo transfer in HRT cycle. 71.4% (n=40) had a positive pregnancy result.

2. All the women with Grade 2 adenomyosis (n=24) were initially given trial of medical management with 2 doses of 11.25 mg GnRH. Out of 24 women, 16 women (66.6%) responded and showed a regression in size of adenomyosis sufficient enough to be taken for transfer. A frozen embryo transfer was planned for such women with medical management and 12 out of 16 women (75%) showed a positive pregnancy test. Rest of the 8 women (33.3%) were taken up for adenomyomectomy in view of failed medical management. Post-surgery GnRH 11.25 mg was also given. 4 out of 8 women (50%) showed a positive pregnancy test.

3. 8 women with diffuse adenomyosis (Grade 3) were initially offered medical management with 11.25 GnRH but they did not respond and had to resort to surrogacy.

4. The remaining 12 patients had Grade 4 adenomyosis with endometriosis and/ or Hydrosalpinx. Out of 12, 5 were treated with 2 doses of GnRH 11.25mg. 4 patients had hydrosalpinx and have to undergo lap hydrosalpinx excision before FET in subsequent cycle. Rest 3 had adenomyoma compressing the cavity with failed medical management and underwent adenomyomectomy with adhesiolysis. Total 8 out of 12 (66.6%) had a positive pregnancy test after frozen embryo transfer in this group.

## DISCUSSION

In literature, there is no consensus over size of adenomyoma which should be medically managed. but based on our study and supporting medical literature, we propose that in a non-cavity distorting adenomyoma less than 4 cm, medical management can improve the pregnancy rates.

In our study, 56% of women were given GnRH 11.25 mg for suppression of HPO axis for 3 months and a frozen embryo transfer was done. It leads to decrease in size of adenomyosis with improved receptivity and implantation. The pregnancy rate in grade1 was 71.4%. It is in concordance with other studies. Lan *et al*. (2021) showed a pregnancy rate of 43% with medical management of adenomyosis but in the study, there was no cut off to categorize which cases are to be medically managed. Similarly, Park *et al*. (2016) reported a pregnancy rate of 39.5% in this group.

In adenomyoma >4cm, management depends on the location of adenomyoma and its distance from the endometrial cavity. When in contact with the cavity, it interferes with adhesion and implantation of embryos and impairs the secretion of various implantation factors. The authors propose that if adenomyoma >4cm but away from cavity, a trial of medical management should be given but in refractory cases or when the adenomyoma is touching the cavity, surgery should be considered. No one particular method of treatment is wholly satisfactory to achieve pregnancy in the nulliparous infertile patient with extensive adenomyosis, and patients should be made to understand this before any treatment is commenced.

In our study, the pregnancy rate was 66.6% in patients with adenomyosis >4cm and were given 3-6 months of GnRh suppression therapy followed by frozen embryo transfer.

The pregnancy rate in group who were given medical treatment but did not respond satisfactorily and were treated with surgery (adenomyomectomy), the pregnancy rate was 33.3%.

Al Jama *et al*. (2011) demonstrated that combined surgical and hormonal treatment had significant benefits with pregnancy rate of 44%. Tsui *et al*. (2015) showed a pregnancy rate of 46% in women treated with adenomyomectomy. Dueholm & Aagaard (2018) showed a pregnancy rate of 47% after surgery in women with adenomyosis.

Uterus-sparing resection of adenomyosis is still an investigational approach especially in patients with extensive adenomyosis who are actively pursuing pregnancy. Adenomyomectomy is done for focal adenomyosis wherein, adenomyoma is separated and excised from the normal myometrial tissue. The technique is similar to myomectomy, although, the plane between normal myometrium and adenomyoma is well defined and the preferred route is laparoscopic adenomyomectomy. The defect is closed with meticulous suturing without leaving any dead space. The best method of surgery is yet to be proven. It was found that, the best minimum wall thickness for excision of the uterus ranges from 9 to 15 mm (Otsubo *et al*., 2016) for conception and preventing uterine rupture during pregnancy, while women with a uterine wall thickness of ≤7 mm may have an elevated risk of subsequent uterine rupture. Thus, wall thickness may be a useful indicator for primary obstetrical management.

It includes diffuse disease which requires long term medical hormonal therapy. However due to extensive anatomical and molecular damage, patients may have to turn to surrogacy as an option. Uterine sparing surgeries should be avoided as much as possible in such cases because the associated risks outweigh the benefits. Otsubo *et al*. (2016) presented results of fertility-saving surgical excisions of diffuse adenomyotic foci and suggested that preservation of a 9 to 15 mm thickness of the uterine wall after excision with classical surgical methods is safe for future pregnancies. But this might not be possible in cases of diffuse adenomyosis. Even when performed by expert surgeons, the rate of uterine rupture in a future pregnancy appears to be 4%. This rate exceeds the uterine rupture rate after myomectomy or classical cesarean birth (2%) (Otsubo *et al*. 2016). Apart from this, there are also increased chances of failed implantation, early abortions and morbidly adherent placenta which can be an obstetrician’s nightmare.

The treatment of such patients is usually surgical. Clipping or excision of hydrosalpinx is an important component in such patients. We performed adhesiolysis with endometrioma excision with bilateral hydrosalpinx excision in these patients and. In our study, the pregnancy rate in this group after surgery was 66.6%. Shi *et al.* (2021) had done a study on similar group of patients and shown a pregnancy rate of 51.8% through IVF after laparoscopic surgery in such patients.

Women with grade I and II A are the candidates who will be significantly benefited from medical management. Unnecessary surgery is thus avoided in such patients reducing cost. Surgery was offered to refractory (to medical management) cases with grade II B and grade IV cases as adenomyoma in such cases was compressing uterine cavity or was associated with dense pelvic adhesion.

The biggest limitation of the current study is the lesser sample size. Hence, further research must be done including larger study population. Also, the primary outcome studied here is the clinical pregnancy rate. The fact that adenomyosis itself is associated with significantly increased risk of abortion cannot be overlooked. This emphasizes need for follow up of these women in order to assess the correlation between the proposed management protocol and the live birth rate.

## CONCLUSION

Our classification is simple and allows cost effective management based on location and extent of disease with the help of ultrasonography. It also helped us to simplify the treatment protocol in patients with adenomyosis and infertility in the unit to achieve desired results.
